# Individual Brain Charting dataset extension, third release for movie watching and retinotopy data

**DOI:** 10.1038/s41597-024-03390-1

**Published:** 2024-06-05

**Authors:** Ana Luísa Pinho, Hugo Richard, Ana Fernanda Ponce, Michael Eickenberg, Alexis Amadon, Elvis Dohmatob, Isabelle Denghien, Juan Jesús Torre, Swetha Shankar, Himanshu Aggarwal, Alexis Thual, Thomas Chapalain, Chantal Ginisty, Séverine Becuwe-Desmidt, Séverine Roger, Yann Lecomte, Valérie Berland, Laurence Laurier, Véronique Joly-Testault, Gaëlle Médiouni-Cloarec, Christine Doublé, Bernadette Martins, Gaël Varoquaux, Stanislas Dehaene, Lucie Hertz-Pannier, Bertrand Thirion

**Affiliations:** 1 Université Paris-Saclay, Inria, CEA, Palaiseau, 91120 France; 2 Department of Computer Science, Western University, London, Ontario Canada; 3 Western Centre for Brain and Mind, Western University, London, Ontario Canada; 4 Criteo AI Labs, Paris, France; 5 FAIRPLAY - IA coopérative: équité, vie privée, incitations, Paris, France; 6 Flatiron Institute, New York, USA; 7 Université Paris-Saclay, CEA, CNRS, BAOBAB, NeuroSpin, 91191 Gif-sur-Yvette, France; 8 Meta FAIR, Paris, France; 9 Cognitive Neuroimaging Unit, INSERM, CEA, Université Paris-Saclay, NeuroSpin center, 91191 Gif-sur-Yvette, France; 10 Collège de France, Paris, France; 11 CEA Saclay/DRF/IFJ/NeuroSpin/UNIACT, Paris, France; 12 UMR 1141, NeuroDiderot, Université de Paris, Paris, France

**Keywords:** Cognitive neuroscience, Databases

## Abstract

The *Individual Brain Charting* (IBC) is a multi-task functional Magnetic Resonance Imaging dataset acquired at high spatial-resolution and dedicated to the cognitive mapping of the human brain. It consists in the deep phenotyping of twelve individuals, covering a broad range of psychological domains suitable for functional-atlasing applications. Here, we present the inclusion of task data from both naturalistic stimuli and trial-based designs, to uncover structures of brain activation. We rely on the *Fast Shared Response Model* (FastSRM) to provide a data-driven solution for modelling naturalistic stimuli, typically containing many features. We show that data from left-out runs can be reconstructed using FastSRM, enabling the extraction of networks from the visual, auditory and language systems. We also present the topographic organization of the visual system through retinotopy. In total, six new tasks were added to IBC, wherein four trial-based retinotopic tasks contributed with a mapping of the visual field to the cortex. IBC is open access: source plus derivatives imaging data and meta-data are available in public repositories.

## Background & Summary

Mapping cognition across the whole human brain requires the multi-dimensional analysis of the correlates of behavior corresponding to a wide range of psychological domains. This phenotyping of behavioral responses relies on brain activation maps obtained from *functional Magnetic Resonance Imaging* (fMRI), that quantify the underlying neural correlates modulated by mental functions across tasks^1–5^.

In order to achieve generalisation of cognitive processes, behavioural phenotyping with fMRI can be carried out through data pooling analyses, in which data from different sources, and therefore different tasks, from different participants in different studies, are aggregated. In this context, it becomes difficult to dissociate effects elicited by: *(1)* common functional signatures of cognitive processes (even if different tasks performed by different participants share some psychological domains); *(2)* within-subject variability in task performance; *(3)* individual functional differences in both between- and within-task performance; and *(4)* different feature distributions across multiple data-acquisition sites.

Inter-subject variability arising from individual functional differences has been widely recognized in neuroimaging and affects all kinds of group-level analyses. It has been shown to undermine not only the estimation of statistical significance^6^, but also the exact demarcation of functional regions according to their contribution in elementary cognitive processes^4^. In the past decade, many studies have thus started to adopt individual analysis, in order to examine both functional and anatomical individual differences^7–13^.

Within-subject variability has been recently addressed in a few studies. They reveal the importance of collecting comprehensive individual data to minimize this type of variability, as it improves—across individuals—task-fMRI replicability measures^14^ and prediction of cognitive phenotypes^4,15^. Additionally, the benefits of increasing the amount of individual data to consequently decrease within-subject variability becomes particularly important in small sample-size regimes^14,15^; this is typically the case of fMRI studies.

Inter-site variability can also affect precision in functional imaging. Multi-site data analyses have become common practice, especially in consortium projects, and technical factors pertaining to scanner platform, imaging sequences and acquisition procedures–including setup of task protocols–have thus been identified as sources of site effects^16^.

On the other hand, cognitive neuroscience has traditionally relied upon event- or block-organized controlled designs using unnatural stimuli^17^. Single-task fMRI experiments are usually conceived in this way, wherein experimental designs tightly control the variables and isolate targeted cognitive constructs as means to link them to the function of a subset of brain regions. Because this approach has been the mainstay in cognitive neuroscience, many of the publicly available task datasets, as well as the ensuing data-pooling studies are mostly based on this type of stimuli^3,17–21^. In contrast, the study of real-life, dynamic and multimodal sensory stimuli–*a.k.a. naturalistic stimuli*–was introduced in parallel in the field of cognitive neuroimaging by two seminal papers^22,23^, and led to initiatives such as the *studyforrest* dataset^24–27^. They are thought to reduce biases inherent to the choice of artificial behavioral settings, and importantly, *a priori* designed contrasts^17^. The main challenges in using naturalistic stimuli include: *(1)* experimental implementation, as naturalistic paradigms are lengthy in duration because their high-dimensional feature space requires the collection of a larger amount of data; *(2)* statistical modelling, as the standard *General Linear Model* (GLM) applied to naturalistic stimuli leads to high-dimensional controlled-design models due to the larger amount of features extracted from the paradigms; and *(3)* unsupervised data-driven approaches are preferred because of *(2)*, but high-dimensional imaging data (many voxels) require decomposition methods that scale.

To obtain a large sample of behavioral features and simultaneously achieve a whole-brain coverage free from inter-site and between-task inter-subject variability, extensive functional mapping at high-spatial resolution in the same set of individual brains exposed to a comprehensive collection of task paradigms is necessary. We thus present herein the third release of the *Individual Brain Charting* (IBC) dataset: a multi-task fMRI-data collection obtained from a permanent cohort of twelve participants acquired with a spatial resolution of 1.5 mm. Its task-wise organization–pertaining to the deep phenotyping of the cohort’s behavioral responses–combined with a higher spatial resolution allows for better estimates of a broad variety of mental functions both at the individual^4,28^ and group level^4,5^.

The first and second releases of the IBC dataset encompass a broad range of cognitive domains, with an emphasis of the second release on higher-order cognition^29,30^. In contrast, the third release is now focused in sensory modalities. It includes two naturalistic tasks on visualization of naturalistic scenes (the *Clips*^31^ task; see Section *Clips* task for more details) and movie watching (the *Raiders*^8^ task; see Section *Raiders* task for more details), probing mainly the visual system (i.e. areas comprising the visual cortex and visual association cortex). Movie watching also covers the auditory system as well as the language system, through inclusion of higher-order components pertaining to speech and narrative comprehension. To improve the coverage of the visual system, we have also included the classic retinotopic tasks^32^ dedicated to map the polar angle and eccentricity in the visual cortex. Table 1 summarizes all tasks from these three releases. Considering our individual functional-atlasing approach based on deep-behavioral phenotyping for cognitive mapping of the human brain, as described in Pinho *et al*.^4^, Thirion *et al*.^5^, and Thirion *et al*.^28^, these tasks are intended to complement those from the first and second releases, through the inclusion of not only existing components but also new ones. Indeed, components concerning visual attention as well as visual object, face and body recognition, already found in *ARCHI Spatial*, *ARCHI Emotional*, *HCP Emotion*, *HCP Relational*, *HCP Working Memory*, *Preference* battery, *Theory-of-Mind* (TOM) battery and *Visual short-term memory* (VSTM) task, are now present in both naturalistic tasks. On the other hand, retinotopic tasks bring new modules on visual representation (i.e. internal representation of visual information; see the Cognitive Atlas^33^), namely functional localization of the meridians and quadrants of the visual field, as well as its foveal, mid-peripheral and far-peripheral areas. Data from the three releases are organized in 31 tasks (see Table 1), of which 29 (the trial-based tasks) amount for 216 contrasts described on the basis of 127 cognitive atoms from the Cognitive Atlas.

**Table 1:** Overview of the IBC tasks from the past and present releases.

Tasks	Description	Release
*ARCHI Standard*	Assess a diversity of mental functions, such as button presses with the left or right hand, viewing horizontal and vertical checkerboards, reading and listening to short sentences, and mental computations.	1
*ARCHI Spatial*	Assess mental functions on spatial cognition, such as the performance of ocular saccade, grasping plus orientation judgment on objects, and left/right plus front/back judgments of hand photographs.	1
*ARCHI Social*	Assess mental functions on social cognition, such as interpretation of false-belief stories, observation of objects with or without putative intentions, and listening to speech and non-speech sounds.	1
*ARCHI Emotional*	Assess mental functions on emotional cognition, such as facial judgments of gender and trustworthiness plus expression based on complete portraits or photos of eyes’ expressions.	1
*HCP*^1^ *Emotion*	Assess affective processing arising from responses to fear and anger.	1
*HCP*^1^ *Gambling*	Assess incentive processing through gambling on the range of the number to be displayed next on the screen.	1
*HCP*^1^ *Motor*	Assess motor capabilities associated with movements of the foot, hand and tongue.	1
*HCP*^1 ^*Language*	Assess semantic processing by listening stories or arithmetic problems (control task).	1
*HCP*^1^ * Relational*	Assess relational-matching mechanisms through a relational matching-to-sample paradigm, featuring a second-order comparison of relations between two pairs of objects.	1
*HCP*^1^ *Social*	Assess social cognition through observation and judgment of objects portraying or not putative social interactions.	1
*HCP*^1^ *Working Memory*	Assess working memory through “2-back” *versus* “0-back” conditions.	1
*RSVP-Language*^2^	Assess syntactic and semantic processing, with additional insights into visual word recognition, sub-lexical processing, and other aspects of active reading.	1
MTT^3^ battery (2 tasks)	Assess the mental time and space shifts involved in the allocentric mapping of fictional events described in terms of audio narratives.	2
*Preference* battery (4 tasks)	Assess decision-making associated with the positive-incentive value and level of confidence in the evaluation of visual constructs.	2
TOM^4^ battery (3 tasks)	Assess theory-of-mind and pain-matrix networks.	2
VSTM^5^ + *Enumeration* (2 tasks)	Assess numerosity with and without encoding of object features.	2
*Self*	Assess the *Self-Reference Effect*.	2
*Bang*	Assess speech comprehension during movie watching.	2
*Clips*	Assess visual processing through visualization of naturalistic scenes.	3
*Retinotopy* (4 tasks)	Map topographic representations of the visual system with the classic retinotopic paradigms.	3
*Raiders*	Assess visual and auditory processing during movie watching.	3

The table contains a short description of every task from the first^29^, second^30^ and third releases, as well as reference to which release each task belongs. All tasks consist in trial-based designs but *Clips* and *Raiders*–from the present release–that are based on naturalistic stimuli.

^1^*HCP*: Human Connectome Project; ^2^*RSVP*: Rapid-Serial-Visual-Presentation; ^3^MTT: Mental Time Travel; ^4^TOM: Theory-of-Mind;

^5^VSTM: Visual Short-Term Memory.

In this paper, we provide a thorough description of the tasks taking part for this extension. We also present their technical validation, using *Quality-Assurance* (QA) metrics that show functional activation to be a direct response to behavior. Additionally and given the aforementioned challenges posed by the analysis of fMRI data relative to naturalistic paradigms, we complement the technical validation of the *Clips* and *Raiders* tasks with an application of the *Fast Shared Response Model* (FastSRM), as described in Richard&Thirion^34^. To this end, we showcase how to extract networks linked to mental functions elicited by these tasks.

IBC is an open-access dataset consisting of high-resolution, functional maps from a dozen of subjects. It aims at providing quantitative insights about individual differences of elementary processes in cognition, by leveraging a deep behavioral-phenotyping approach. Many sessions pertaining to different tasks are thus undertaken per participant. Unlike longitudinal studies, data collection of each task only takes place once during the lifetime of the project. IBC is thus intended to serve as a source of functional correlates of various cognitive conditions, in order to support research in human neuroscience.

## Methods

To avoid ambiguity with MRI-related terms, definitions follow the *Brain-Imaging-Data-Structure* (BIDS) Specification version 1.8.0^35^.

### Participants

The present release of the IBC dataset consists of brain fMRI data from twelve individuals (two female) acquired between April 2016 and February 2019. The experiments were carried out with the understanding and formal consent of the participants, in accordance with the ethical principles of the Helsinki declaration, as well as the French public health regulation (https://ansm.sante.fr/), which approved the study protocol.

Detailed description of the age, sex and handedness of the group is provided in Table 2. Age varied between 26 and 40 years old (median = 30 years) upon recruitment and handedness was determined with the *Edinburgh Handedness Inventory*^36^. For more information on the cohort’s recruitment, consult Pinho *et al*.^29^ and Pinho *et al*.^30^.

**Table 2:** currentlabel1Demographic data of the participants.

Subject ID	Year of recruitment	Age	Sex	Handedness score
sub-01	2015	39.5	M	0.3
sub-04	2015	26.9	M	0.8
sub-05	2015	27.4	M	0.6
sub-06	2015	33.1	M	0.7
sub-07	2015	38.8	M	1
sub-08	2015	36.5	F	1
sub-09	2015	38.5	F	1
sub-11	2016	35.8	M	1
sub-12	2016	40.8	M	1
sub-13	2016	28.2	M	0.6
sub-14	2016	28.3	M	0.7
sub-15	2017	30.3	M	0.9

Age stands for the participants' age upon recruitment.

### Materials

#### Stimuli

This release covers data from three main behavioral protocols: *Clips* task, *Retinotopy* battery of tasks (i.e. *Wedge* and *Ring* tasks), and *Raiders* task (see Experimental Paradigms Section for details about tasks' paradigms). The stimuli of the tasks were delivered through custom-made scripts that ensured a fully automated environment and computer-controlled collection of the behavioral data. All protocols were set under Python 2.7. The protocol of the *Clips* task was adapted from the original study^31^ using standard Python libraries; the ones pertaining to the *Retinotopy* and *Raiders* tasks were respectively designed with *PsychoPy* v1.90.3^37^ and *Expyriment* v0.9.0^38^. Visual stimuli of *Clips* consisted of the same color natural movies as described in Nishimoto *et al*.^31^, whereas the audiovisual stimuli presented in the *Raiders* task corresponded to the 2009 DVD edition of *Raiders of the Lost Ark* dubbed to French.

These materials are available in a public GitHub repository dedicated to the behavioral protocols of the tasks featuring the IBC dataset: https://github.com/individual-brain-charting/public_protocols (consult Section Code Availability for further details about the repository).

#### Eye-tracker

The video-based, eye-tracker system *EyeLink 1000 Plus* was used for the behavioral, training sessions of the *Clips* plus *Retinotopy* tasks (for more information about the training sessions, consult Section Experimental Procedure).

#### MRI equipment

The fMRI data were acquired using an MRI scanner Siemens 3 T Magnetom Prismafit along with a Siemens Head/Neck 64-channel coil. Behavioral responses for the *Retinotopy* tasks were obtained with a MR-compatible, five-button ergonomic pad (Current Designs, Package 932 with Pyka HHSC-1 × 5-N4) and the MRI-environment audio system for the *Raiders* task was set with the MR-Confon package.

All sessions were conducted at the NeuroSpin platform of the CEA Research Institute, Saclay, France.

### Experimental procedure

Upon arrival to the research institute, participants were instructed about the execution and timing of the tasks referring to the upcoming session.

Particularly, behavioral training sessions prior to the MRI sessions were conducted for the *Clips* and *Retinotopy* tasks. We stress that the center of the visual field must be approximately constant during data acquisition of these tasks, as means to obtain a consistent map of the visual system within and between individuals (consult Experimental Paradigms Section for more details). To this end, participants were prepared during the training sessions to gain perception of their eyes' movements. They were instructed to move them as little as possible while fixating toward a flickering point placed on the center of a screen, which was also displaying video scenes at the same time. These video scenes were excerpts of those presented for the *Clips* task. An eye-tracker was also coupled to the experimental setup of the training session. Participants were thus provided with a real-time feedback of their eyes' movements in the form of a green moving point displayed on the screen, too. The main goal of this training exercise was to keep the green moving point as close as possible to the flickering one, which was fixed. By this way, participants could then practice how to keep looking continuously toward the center of the screen—i.e. the perimetric origin—for as long as possible.

All MRI sessions were composed of several runs dedicated to one or two tasks as described in Section Experimental Paradigms. The structure of the sessions according to the MRI modality employed at every run is detailed in Table 3. Information on the imaging parameters of the referred modalities can be found in Imaging Data Section.

**Table 3:** currentlabel2Plan of the MRI acquisitions for the third release of the IBC dataset.

Session	Modality	Task	Duration of each Run* (min:sec)	Repetitions
Clips 1–3	2D Spin-Echo	—	00:31	PA(×2) + AP(×2)
	BOLD fMRI	Clips training set	10:50	PA(×2) + AP
	BOLD fMRI	Clips test set	10:50	PA + AP(×2)
Clips 4	2D Spin-Echo	—	00:31	PA(×2) + AP(×2)
	BOLD fMRI	Clips training set	10:50	PA(×2) + AP
	BOLD fMRI	Retinotopy wedge clock	05:30	PA(×2) + AP(×2)
	BOLD fMRI	Retinotopy wedge anti-clock	05:30	PA(×2) + AP(×2)
	BOLD fMRI	Retinotopy ring expanding	05:30	PA
	BOLD fMRI	Retinotopy ring contracting	05:30	AP
Raiders 1	2D Spin-Echo	—	00:31	PA(×2) + AP(×2)
	BOLD fMRI	Raiders chapter 1	12:28	PA
	BOLD fMRI	Raiders chapter 2	09:53	PA
	BOLD fMRI	Raiders chapter 3	10:28	PA
	BOLD fMRI	Raiders chapter 4	12:37	AP
	BOLD fMRI	Raiders chapter 5	11:33	AP
	BOLD fMRI	Raiders chapter 6	11:32	AP
Raiders 2	2D Spin-Echo	—	00:31	PA(×2) + AP(×2)
	BOLD fMRI	Raiders chapter 7	11:39	PA
	BOLD fMRI	Raiders chapter 8	11:46	PA
	BOLD fMRI	Raiders chapter 9	09:22	AP
	BOLD fMRI	Raiders chapter 10	07:01	AP
	BOLD fMRI	Raiders chapter 1	12:28	AP
	BOLD fMRI	Raiders chapter 2	09:53	AP
	BOLD fMRI	Raiders chapter 3	10:28	AP

A BOLD-fMRI run refers to the acquisition of fMRI data on one single task. At least, there were two BOLD runs, corresponding to PA- and AP- phase-encoding directions for each task during a session. The 2D Spin-Echo PA/AP volumes were always acquired before the runs dedicated to the collection of BOLD-fMRI data and repeated afterwards.

*For BOLD fMRI sequences, the durations herein presented account only for the period of the actual acquisition, included in the data volumes. The full duration of each run also included ∼45 s of dummy scans, always performed at their beginning.

### Experimental paradigms

Tasks were aggregated in different sessions according to Table 3. The following sections intend to provide a description of the paradigms employed for each task. Materials used for stimulus presentation (see Section Stimuli) have been made publicly available, together with video-demo presentations of the corresponding protocols, on https://github.com/individual-brain-charting/public_protocols (see Section Code Availability for a detailed description of the repository).

A comprehensive explanation of the experimental paradigms as well as conditions and contrasts of the *Retinotopy* tasks can be also found on the online documentation of the IBC dataset: https://individual-brain-charting.github.io/docs.

#### Clips task

The *Clips* task stands for a reproduction of the study reported in Nishimoto *et al*.^31^, in which participants were to visualize naturalistic scenes edited as video clips of ten and a half minutes each.

Data collection was carried out in uninterrupted runs, each of them dedicated to the presentation of a specific video clip. As in the original study, runs were grouped in two categories: training and test. Scenes from the training category were shown only once in the corresponding video clip. Contrariwise, scenes from the test category were composed of approximately one-minute-long excerpts selected from the clips presented during training. Excerpts were concatenated to construct the sequence of every test run; each sequence was predetermined by randomly permuting many excerpts that were repeated ten times each across all runs. The same randomized sequences, employed across test runs, were used to collect data from all participants.

There were twelve and nine runs dedicated to the collection of training data and test data, respectively. Data from nine runs of each category were interspersedly acquired over three full sessions. The three remaining runs for training-data collection were acquired in half of one last session, prior to the *Retinotopy* tasks (see Section *Retinotopy* tasks for complete description of these tasks).

To assure the same topographic reference of the visual field for all participants, a colored fixation point was always presented at the center of the images. Such point was changing three times per second to ensure that it was visible regardless the color of the movie. To account for stabilization of the BOLD signal, ten extra seconds of acquisition were added at the beginning and end of every run. The total duration of each run was thus ten minutes and fifty seconds.

#### Retinotopy tasks

The *Retinotopy* tasks refer to the classic retinotopic paradigms—i.e. the *Wedge* and the *Ring* tasks—consisting of four kinds of visual stimuli: *(1-2)* a slowly rotating clockwise or counterclockwise, semicircular checkerboard stimulus, as part of the *Wedge* task; and *(3-4)* a thick, dilating or contracting ring, as part of the *Ring* task. The phase of the periodic response at the fundamental frequency of rotation or dilation/contraction measured at each voxel is related to the measurement of the perimetric parameters of *polar angle* and *eccentricity*, respectively^32^.

In the present study, six runs were devoted to this task. Each of them were five-and-a-half minutes long. They were programmed for the same session following the last three “training-data” runs of the *Clips* task (see Section *Clips* task for complete description of this task). Four runs were dedicated to the presentation of the rotating checkerboard stimulus (two runs for each direction) and the remaining two were dedicated to the dilating or contracting ring, one at a time.

Similarly to the *Clips* task, a point was displayed at the center of the visual stimulus in order to keep constant the perimetric origin in all participants. Participants were thus to fixate continuously this point whose color flickered throughout the entire run. To keep the participants engaged in the task, they were instructed that, at the end of every run (i.e. after MRI acquisition was finished), they would be asked which color had most often been presented. They had to select one of the four possible options–i.e. red, green, blue or yellow–by pressing on the corresponding button in the response box.

#### Raiders task

The *Raiders* task was adapted from Haxby *et al*.^8^, in which the full-length action movie *Raiders of the Lost Ark* was presented to the participants. The main goal of the original study was the estimation of the hyperalignment parameters that transform voxel space of functional data into feature space of brain responses, linked to the visual characteristics of the movie displayed.

Similarly to the *Clips* task, the movie was shown herein to the IBC participants in contiguous runs determined according to the chapters of the movie defined in the DVD (see Section Stimuli for details about the DVD edition). Yet, one shall note that while the raiders task involved free-viewing, the *Clips* task required fixation.

This task was completed in two sessions. In order to use the acquired fMRI data in train-test split and cross-validation experiments, we performed three extra-runs at the end of the second session in which the three first chapters of the movie were repeated.

To account for stabilization of the BOLD signal, ten seconds of acquisition were added at the end of the run. The structure of both sessions and duration of their runs are detailed in Table 3.

### Data acquisition

Data across participants were acquired throughout four MRI sessions, whose structure is described in Table 3. Deviations from this structure was only registered for participant 8 (sub-08), whose data pertaining to Run #13 of *Raiders* task was acquired in the session dedicated to the *Theory-of-Mind* and *Pain Matrices* task battery from the second release of the IBC dataset (for more details about this release, consult Pinho *et al*.^30^).

#### Behavioral data

Behavioral data from the training sessions for the *Clips* and *Retinotopy* tasks were recorded throughout four sessions.

Scores were obtained according to the response accuracy (in percentage) of the participant for a given trial. They indicate how close participant’s sight was from the center of the screen during the trial. Each session was composed of four different trials. Therefore, we collected a maximum of sixteen scores per participant across all sessions. They are presented in the Supplementary Material. Each participant completed at least one training session. However, the completion of the four sessions was not mandatory, as there was a trade-off between extensive training and quality of performance. Since participants could experience some fatigue while fixating their eyes for a continuous period of time, we thus recommended carrying out at least one training session (for more information about how training sessions were conducted, consult Section Experimental Procedure). We thus denote that the recorded scores were merely intended to facilitate participants' training and no further analyses were conducted with these data.

Additionally, participants were also asked to provide a button-press response at the end of every run concerned with the *Retinotopy* tasks (for more details about these tasks, consult Section *Retinotopy* tasks). The registry of these behavioral data was held in log files generated by the stimulus-delivery software and the response accuracy across runs for each participant is provided in Section Behavioral Results.

#### Imaging data

FMRI data were collected using a *Gradient-Echo* (GE) pulse, whole-brain *Multi-Band* (MB) accelerated^39,40^ *Echo-Planar Imaging* (EPI) T2*-weighted sequence with *Blood-Oxygenation-Level-Dependent* (BOLD) contrasts, using the following parameters: the repetition time (TR) is 2000 ms; the echo time (TE) is 27 ms; the flip angle is 74°; the field-of-view (FOV) is 192 × 192 × 140 mm^3^; voxel size is 1.5 × 1.5 × 1.5 mm^3^; the slice orientation is axial; slices are acquired in interleaved fashion; in-plane acquisitions were accelerated by a factor (GRAPPA) of 2; and across slices, a multi-band factor of 3 was used. Two different acquisitions for the same task were always performed using two opposite phase-encoding directions: one from *Posterior to Anterior* (PA) and the other from *Anterior to Posterior* (AP). The main purpose was to mitigate geometrical distortions while assuring built-in, within-subject replication of the same tasks.

*Spin-Echo* (SE) EPI-2D image volumes were acquired in order to correct for spatial distortions, using the following parameters: a TR of 7680 ms; a TE of 46 ms; a FOV of 192 × 192 × 140 mm^3^; a voxel size of 1.5 × 1.5 × 1.5 mm^3^; axial slice orientation; and acceleration factor (GRAPPA) = 2. Similarly to the GE-EPI sequences, two different acquisitions were also performed using PA and AP phase-encoding directions.

In addition, a *3D magnetization-prepared rapid gradient-echo* (MP-RAGE) T1-weighted anatomical-image volume, covering the whole brain, was acquired with the following parameters: voxel size of 1 × 1 × 1 mm^3^; sagittal slice orientation; flip angle of 9°; and FOV of 256 × 256 × 160 mm.

A detailed description of the imaging parameters set for each MRI modality is available in Pinho *et al*.^29^ and in the IBC-dataset documentation: https://individual-brain-charting.github.io/docs.

### Imaging-Data analysis

Prior to any neuroimaging-data analysis, the MRI DICOM images were converted to NIfTI format using the *DCM2NII* tool, which is available on https://www.nitrc.org/projects/dcm2nii. Conversion to NIfTI format also included a full anonymization of the data, i.e. pseudonyms were removed and images were defaced using the Freesurfer-6.0.0 library^41^.

#### Preprocessing

All GE-EPI volumes (*aka* the functional volumes) were collected twice with reversed phase-encoding directions, resulting in pairs of images with distortions going in opposite directions. Susceptibility-induced off-resonance field was estimated from the two SE-EPI volumes, which were collected twice and also using reversed phase-encoding directions. The GE-EPI volumes were then corrected based on the corresponding deformation model, which was computed using the FSL implementation as described in Andersson *et al*.^42^ and Smith *et al*.^43^.

Source data were then preprocessed using the same pipeline as described in Pinho *et al*.^29^ and Pinho *et al*.^30^, which relies on the *PyPreprocess* library: https://github.com/neurospin/pypreprocess. Pypreprocess stands for a collection of Python scripts–built upon the *Nipype* interface^44^–which are oriented toward a common workflow of fMRI-data preprocessing analysis; it uses precompiled modules of both *SPM12* software package (Wellcome Department of Imaging Neuroscience, London, UK) and *FSL* library (Analysis Group, FMRIB, Oxford, UK) v6.0.0.

GE-EPI volumes of each participant were aligned among them^45^; the mean EPI volume was co-registered onto the corresponding T1-weighted MPRAGE volume (*aka* the anatomical volume); the individual anatomical volumes were segmented for the estimation of the deformation field to be applied on the normalization of both anatomical and functional volumes^46^.

The T1-weighted MPRAGE volume and the aligned GE-EPI volumes were then given as input to *FreeSurfer* v6.0.0, in order to extract the surface meshes of the tissue interfaces and a sampling of the functional activation on these meshes, as described in vanEssen *et al*.^47^. These functional activations were then resampled onto the fsaverage7 template of FreeSurfer^48^.

For more information about the IBC preprocessing pipeline, consult ‘Preprocessing' section of Pinho *et al*.^29^ and/or the online documentation of the IBC dataset: https://individual-brain-charting.github.io/docs.

#### Naturalistic-Data analysis

Naturalistic stimuli typically imply a profuse amount of data descriptors, which leads to high-dimensional design matrices^49,50^. Therefore, a data-driven approach was instead employed herein as means to capture the underlying structure of the fMRI data and the ensuing effects-of-interest elicited by this type of behavioral paradigms.

Because brains exposed to the same stimuli exhibit synchronous activity^23^, a shared response can be obtained across different individuals. To this end, we used both a model-free and a model-based approaches.

*Inter-Subject Correlations* (ISC)^23^ of individual preprocessed data (see Section Preprocessing) can naturally capture this common response without explicitly model the data. For each run, we thus calculated the Pearson correlation across all voxels for every pair of subjects. We then calculated the mean of the pairwise correlation of each run and subsequently across runs (for results regarding this analysis, refer to Fig. 3).

Nevertheless, one disadvantage that arises from ISC is the fact it misses correspondences that are not accurate at the voxel level. We used the FastSRM implementation for fMRI data–as described in Richard&Thirion^34^–to analyze the preprocessed imaging data of *Clips* and *Raiders* and extract a common subjects' response to these tasks together with their individual spatial components. These individual spatial components thus refer to the subject-specific effects-of-interest elicited by the tasks. To be able to statistically validate these findings, we set an analysis based on *Cross Validation* (CV), in which we assessed whether reconstructed individual data of one run predicted the original individual data for that run. As referred in Table 3, data per participant and for one task were collected in different runs during one or more sessions and, consequently, data from one run pertain only to one task. Moreover, *Raiders* and *Clips* data were analyzed separately. By this way, this prediction framework is task specific.

Concretely, it consists in a double K-fold cross validation based on *co-smoothing*^51^, wherein *K* = 3 for *N* = 12 subjects and *K* = 2 for *R* runs. The total number of runs per task is different. For *Raiders*, *R* = 13 and, therefore, the size of the two folds are six and seven. For *Clips*, data were split in terms of number of runs for training and test, which were predefined according to the acquisition design of the task (see *Clips* task for more details); the size of the training and test sets are thus twelve and nine, respectively. For the two tasks, each fold comprised runs with both phase-encoding directions. This CV procedure served therefore as a scoring algorithm. It computes the correlation coefficient between predicted brain-activity maps of test runs and subjects–which were obtained from the shared response and *k* = 20 spatial components learnt on the training runs–and the corresponding original data. The resulting vertex-wise correlation coefficients represent a similarity measure of the shared content between subjects. This procedure is depicted in further detail on Fig. 4. To compute the group-level significance of these individual estimates, we obtained p-values through a mass-univariate group analysis and corrected for multiple comparisons, using the *False Discovery Rate* (FDR); subsequent vertex-wise *q*–*values* ≤ 0.05 served as a mask to display the group-level correlation coefficients (for results regarding this analysis, refer to Fig. 5b). To evaluate the upper-bound model performance of FastSRM, we determined the noise ceiling using data from the three first runs of session 1 of the *Raiders* task. Because these runs were repeated at the end of session 2, the noise ceiling was estimated, for every subject, as the vertex-wise correlation between the same run and its repetition. The correlation between original data and reconstructed data of the same run was thus expressed as a fraction of this noise ceiling. We then took the vertex-wise median of these ratios across subjects, normalized them to ensure they lie between 0 and 1 and, finally, took the median across runs (for results regarding this analysis, refer to Fig. 5a).

We also assessed what regions are significantly different in the performance of *Raiders versus Clips* by computing a two-sided paired *t*-test in every vertex, between the individual correlation coefficients of the two tasks (for results regarding this analysis, refer to Fig. 5c).

In order to obtain a precise labeling of the functional regions covered by the previous results, we computed the proportion of significant vertices present in the areas and regions derived from the cortical parcellation of Glasser *et al*.^52^. This estimation was performed using the projection of the HCP-MMP1.0 parcellation onto the fsaverage7 template, which is available on Mills (2016)^53^ (for results regarding this analysis, refer to Supplementary Table 1).

The FastSRM encoding analysis was implemented using the *IdentifiableFastSRM* module of the FastSRM package that can be found on: https://github.com/hugorichard/FastSRM. Statistical analyses and plotting were performed using *SciPy* v1.6.1 (https://www.scipy.org) and *Nilearn* v0.9.2^54^ (https://nilearn.github.io).

#### Retinotopic-data analysis

The retinotopic mapping data (rotating wedge and expanding/shrinking rings) were analyzed using the standard frequency-domain analysis described in Sereno *et al*.^32^ and Warnking *et al*.^55^: sine/cosine regressors were specified at the frequency of the periodic stimulus position change ($\frac{1}{32}Hz$). The magnitude of the reponse of the voxels was estimated across six sessions, and tested for significance using an *F*– test, thresholded at *p* < 0.001 and uncorrected for multiple comparisons. The phase of the reponse in each voxel with a significant effect was then determined for each session, by comparing the relative magnitude of the sine and cosine regressors. The phase information was combined for wedge- and ring-stimuli separately, in order to cancel the hemodynamic-induced phase delay. This is possible because the stimuli were presented in opposite motions (clockwise *versus* anti-clockwise for the wedge and expanding *versus* contracting for the ring). In addition, results were averaged across replications (wedge experiments). The phase estimate therefore defines in polar coordinates (eccentricity and polar angle) the visual field position that elicits a maximal amount of activation in each voxel.

All these steps were computed on the data sampled on the *fsaverage7* template using the Freesurfer software^48^. For visualization, the surface-based polar angle and eccentricity maps were displayed using the flat representation available through the Pycortex tool^56^.

## Data Records

Source and derivatives data of the IBC dataset are accessible online to the public. Derivatives account for both preprocessed imaging data (i.e., normalized functional and anatomical data; for detailed information about preprocessing, consult Section Preprocessing) and the subsequent postprocessed imaging data. Postprocessed derivatives refer specifically to the individual and unthresholded *z*-maps, that are obtained from the contrast maps of the experimental conditions pertaining the trial-based tasks. Therefore, for the present release, these derivatives are only available for the *Retinotopy* tasks (see Section Retinotopy Study).

The online access of the data is assured by the *Human Brain Project* (HBP) EBRAINS platform^57^ under the project *Individual Brain Charting (IBC) v3.0*^58^. It is also possible to use an integrated data-fetcher API tool to download wholly or partially the IBC dataset, which includes not only the third release but also other releases not herein reported. For more information on how to install and use the data fetcher, consult the IBC documentation available online: https://individual-brain-charting.github.io/docs. For more information about the API tool, consult Section Code Availability.

Source data is also available in the OpenNeuro public repository^59^, under the accession number *ds*002685^60^, and postprocessed derivatives can be found in the *NeuroVault* repository^61^, under the collection with the id = 6618^62^.

The NIfTI files as well as paradigm descriptors and imaging parameters are organized per run for each subject and session, according to BIDS Specification, in both EBRAINS and OpenNeuro. For more details, consult ‘Data Records' sections of Pinho *et al*.^29^ and Pinho *et al*.^30^: the data descriptors of the IBC first and second releases, respectively.

## Technical Validation

Behavioral results were obtained through an elementary assessment of curated behavioral data. They are reported in Section Behavioral Results.

All imaging results were obtained following the methodological procedures, as presented in Sections Naturalistic-Data Analysis and Retinotopic-Data Analysis, applied to task-fMRI data previously preprocessed on the surface, as described in Section Preprocessing. They are respectively reported in Sections FastSRM-Encoding Study and Retinotopy Study.

### Behavioral results

In the *Retinotopy tasks*, participants were to give a button-press response at the end of every run corresponding to the color of the flickering fixation point they saw most times. There was only one correct answer out of the four possible options provided for each task run, which was fixed across participants. Table 4 displays the individual response accuracy achieved for all task runs. These scores are presented as percentages of the individual correct responses with respect to the total number of (correct) responses; because there were six runs dedicated to the *Retinotopy* tasks (see Table 3), six responses were obtained. The average ± standard deviation of the response accuracy across participants (excluding participant 5) are 56 ± 20%, i.e. higher than chance level (25%). These results show that overall participants' fixation was good enough to perform a discrimination task during the course of the run, thus suggesting that fixation was held properly.

**Table 4:** currentlabel3Response accuracy (%) of behavioral performance for the *Retinotopy* tasks.

Subject ID	Response Accuracy (%)
sub-01	83
sub-04	67
sub-05	—
sub-06	50
sub-07	33
sub-08	67
sub-09	67
sub-11	67
sub-12	33
sub-13	33
sub-14	33
sub-15	83
Chance level: 25%	

Scores were estimated based on the correct answers of each participant across the six *Retinotopy* runs, i.e. comprising both the *Wedge* and the *Ring* tasks. The absence of response accuracy for subject 05 relate to loss of behavioral data due to a transient malfunctioning of the equipment; we stress that this issue refers to a misregistration of the data in the log files generated by the stimulus-delivery software and, thus, agnostic to subjects' performance.

### Imaging results

#### Data quality

**Fig. 1:**
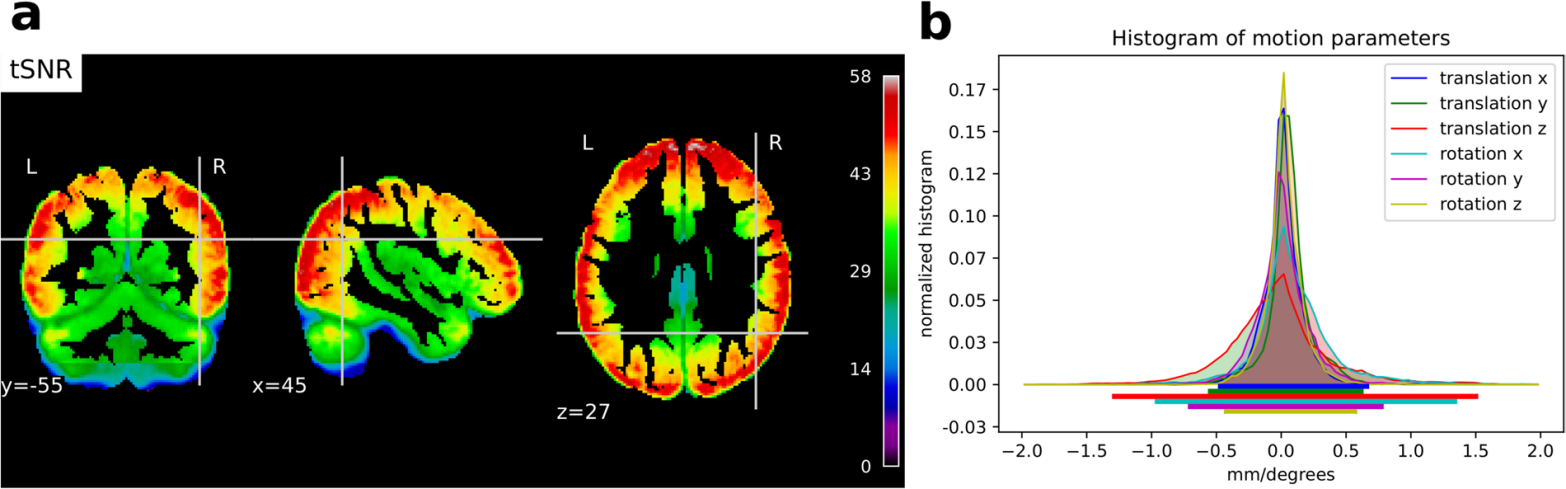
Global quality indices of the acquired data: tSNR map and motion magnitude distribution. (**a**) The tSNR map displays the average of tSNR across all tasks and subjects in the gray matter. This shows values mostly between 30 and 58, with larger tSNR in cortical regions. (**b**) Density of within-run motion parameters, pooled across subjects and tasks. Six distributions are plotted, for the six rigid-body parameter of head motion (translations and rotations are in mm and degrees, respectively). Each distribution is based on ∼145k EPI volumes of 12 subjects, corresponding to all time frames for all acquisitions and subjects. Bold lines below indicate the 99% coverage of all distributions and show that motion parameters mostly remain confined to 1.5 mm/1degree across 99% of all acquired images.

**Fig. 2:**
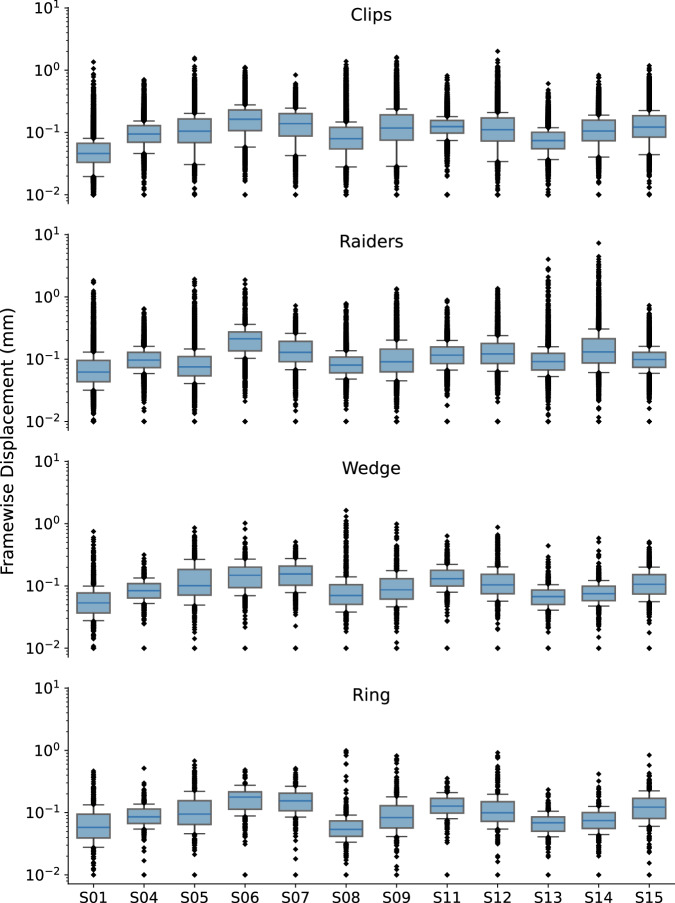
Distributions of the individual framewise displacements, in mm, across runs of each task. Framewise displacement is expressed as a scalar of the instantaneous head motion, that is described by the six rigid body-motion estimates. To facilitate visualization, the vertical axis is represented in the logarithmic scale and framewise-displacement values lower than 0.01 mm were clipped.

In order to provide an approximate estimate of data quality, standard QA measures are presented in Figs. 1 and 2 according to:

- The *temporal Signal-to-Noise ratio* (tSNR), defined as the mean of each voxels' time course divided by their standard deviation, on normalized and unsmoothed data averaged across all acquisitions (from all subjects). Its values are ≥ 50 in most cortical regions. Given the high resolution of the data (1.5 mm isotropic), such values are indicative of a good image quality^63^;
- The histogram of the six rigid body-motion estimates of the brain per scan, in mm/degree, together with their 99% coverage interval. One can notice that this interval ranges approximately within [−1,1] mm/degree, showing that excursions beyond 1 mm/degree motion are rare. No acquisition was discarded due to excessive motion (>2 mm/degree);
- The framewise displacement, computed as described in Power *et al*.^64^. This scalar quantity, estimated from the six rigid body-motion estimates across runs, displays similar distributions for every subject and task. Median of individual displacements is usually lower than 0.1 mm and their upper quartile never goes beyond 0.25 mm.

**Fig. 3:**
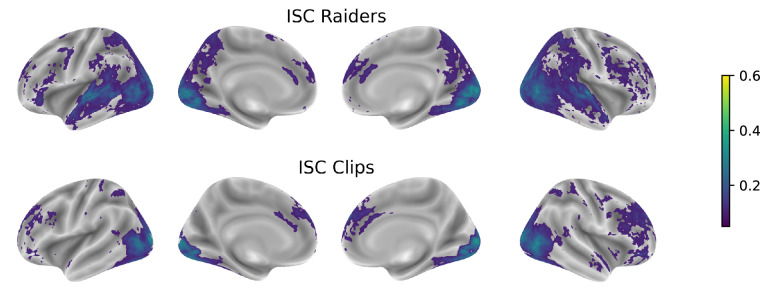
Inter-subject correlation maps for *Raiders* and *Clips* tasks. Pearson-correlation coefficient values were estimated as the mean across runs of the means, obtained for every run, of the pairwise inter-subject correlations of the individual GE-EPI data preprocessed on the surface. For visualization purposes, the maps were thresholded to 0.05. Estimations were performed separately for each task.

Since brain activation measured with fMRI during naturalistic stimuli can be expected to consist largely of stimulus-driven, between-subject effects shared by the majority of participants, ISC of individual fMRI time series thus provide a summary statistic of subject synchrony over the entire imaging session. Figure 3 shows where positive inter-subject correlations of BOLD signal are mapped for each task across the brain and highlights the striking similarity of these representations with those obtained from the individual, model-based approach (see Fig. 5b). One can thus observe that spatial locations wherein brain activation is more similar across subjects are also those that likely reflect a significant response to behavior.

**Fig. 4:**
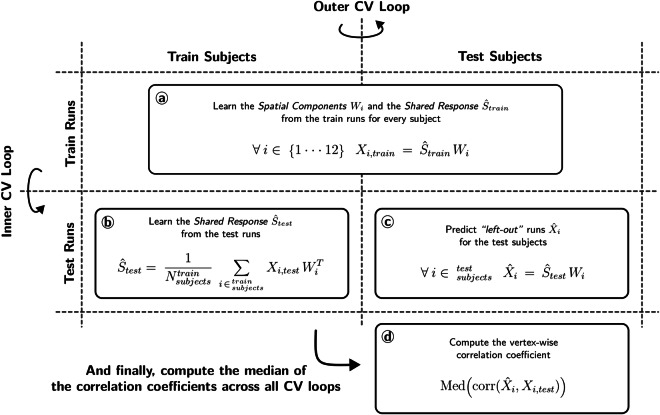
Description of the co-smoothing procedure to compute the jointly activated brain areas using FastSRM. The algorithm runs two nested CV loops. One first outer loop executes a random split of the cohort of twelve participants into a train set and a test set, respectively composed of eight and four subjects. One inner loop executes a random split of the group of runs into a train set and a test set: respectively twelve and nine for *Clips* and, interchangeably seven or six for *Raiders*. For every turn of the nested CV loops: (**a**) the *k* = 20 spatial components specific to each subject on the train runs are computed through alternate minimization together with their shared response, which is then used to compute the individual components of test subjects on the same runs; then, (**b**) assuming that the same features of the train runs will be found on the test runs, we fit the individual responses of the train subjects on the test runs in order to compute their shared response; (**c**) test runs are then predicted through their shared response computed in (**b**); and, (**d**) the vertex-wise correlation between the predicted runs and the corresponding original data is computed. For every subject, we estimated the vertex-wise median of the correlations across runs. The vertex-wise median of the correlations across all subjects was then estimated from their individual median correlations. This final coefficient represents the similarity of activated regions across subjects for each task.

**Fig. 5:**
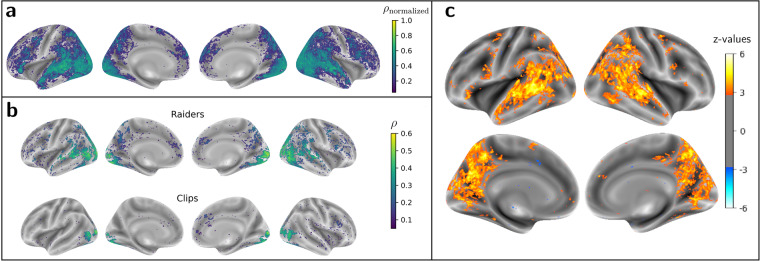
Statistical validation of naturalistic-stimuli tasks with FastSRM. (**a**) Person-correlation coefficients (*ρ*_*normalized*_) of FastSRM prediction for *Raiders* task, compared to noise ceiling. The noise ceiling was estimated as correlations between run pairs 1-11, 2–12 and 3–13 that refer to the same video excerpts. The correlation between runs 1, 2 and 3 with their reconstructed runs from FastSRM are expressed as a fraction of this noise ceiling. These ratios were computed for every vertex and subject. We then took the median across subjects, normalized and took the median across runs. Results are thresholded at 0.1. **(b)** Group-level, Pearson-correlation coefficients (*ρ*) between the original and reconstructed data for *Raiders* and *Clips* tasks. Coefficients were obtained for every vertex from a double *K*–fold cross-validation experiment across subjects (*K* = 3) and runs (*K* = 2) for each task. Data of test subjects performing test runs were reconstructed from the projection of the shared response of train subjects while performing test runs onto the individual components of test subjects while performing train runs. Predictions between original and reconstructed data were estimated for every subject and run. To obtain the group-level estimation of the coefficients, the vertex-wise median of the coefficients were firstly taken within split-halfs, secondly between split-halfs for every subject, and finally across subjects. To assess the group-level significance of these estimates, we computed a mass-univariate non-parametric analysis, then derived an FDR-corrected p-value. Coefficients are only displayed for vertices with *q* ≤ 0.05. **(c)** Group-level *z*–maps displaying brain activation significantly different between *Raiders* and *Clips* tasks. Results were determined through a vertex-wise paired *t*–test between the individual Pearson-correlation coefficients of the two tasks and standardized afterwards. Statistical significance was assessed using an FDR-corrected threshold *q* = 0.05. Clusters depicted in orange/yellow represent brain activation significantly higher for *Raiders* than *Clips*, whereas those depicted in dark/light blue represent brain activation significantly higher for *Clips* than *Raiders*. Orange-yellow clusters surpass in number and size the blue clusters, highlighting the additional cognitive recruitment in *Raiders* related to auditory and language comprehension specific to this task.

Figure 4 describes the procedure implemented to validate naturalistic-stimuli data using FastSRM. It estimates the jointly activated brain areas, in which inference is performed in a two-nested CV loop. Two core assumptions underlie this approach: *(1)* “Step a” of Fig. 4 assumes the same shared response *S* for all subjects relative to the same task; and *(2)* “Step b” assumes that features between similar tasks share the same within-subject representation denoted by *W*_*i*_. We also investigated the between-subject noise ceiling of FastSRM using data pertaining to six runs of the *Raiders* task. Figure 5a shows that FastSRM could account for half of the noise ceiling in terms of correlation, in occipital, dorsal and temporal regions.

#### FastSRM-encoding study

Figure 5b shows the measures of performance—across subjects—of the FastSRM algorithm in terms of Pearson-correlation coefficients (see Section Naturalistic-Data Analysis and Fig. 4 for details), which were obtained between predicted and original data. These results yield functional regions that are synchronously activated across subjects for each task, because predicted data is herein estimated from a shared response to the same stimuli—learnt from the model—which is assumed to be observed in all individuals.

Higher significance was obtained throughout occipital areas for both *Raiders* and *Clips* tasks. Overall, these activations suggest a prominent recruitment of areas pertaining to the visual system^52,65^ during performance of both *Raiders* and *Clips* tasks. These areas comprise both early visual areas and middle temporal regions, including the *MT* + *Complex*, as well as associative visual areas from the dorsal and ventral streams^66^.

Similar activations were also found in the *Inferior Parietal Lobe* and posterior areas of the *Superior Temporal Sulcus* only for the *Raiders* task, indicating the additional recruitment of the auditory and language-comprehension systems during performance of this task. They also reflect the main behavioral differences underlying *Clips* and *Raiders* tasks, i.e. they highlight the fact that while both tasks refer to naturalistic visual stimuli, only *Raiders* refers to naturalistic audio stimuli.

To obtain a clear distinction of the regions exhibiting greater contributions for *Raiders* than *Clips* and vice-versa, we further inspected the *z*– maps from Fig. 5c depicting clusters whose difference of activations between the two tasks is significant. The results show a large number of regions where the activation level for *Raiders* is significantly higher than for *Clips*, while the extent of regions showing results in the opposite direction is extremely small. The identification of the functional territories covered by the clusters was determined in agreement with the parcellation of Glasser *et al*.^52^, which comprises 180 neocortical areas that are subsequently grouped in 22 main regions. Supplementary Table 1 presents, by descending order, the list of areas that contain a proportion of significant vertices larger than 5% in both hemispheres. The correspondence between area and main region was established according to the primary section in which the given area is described by the cortical parcellation (for further details, consult Table 1 of the *Neuroanatomical Supplementary Results of* Glasser *et al*.^52^). We identified 94 areas belonging to 21 main regions displaying higher activation for *Raiders* than *Clips*; no areas displaying higher activation for *Clips* than *Raiders* were observed above the same threshold. These results confirm the recruitment of additional brain networks necessary in the performance of cognitive tasks—namely auditory, speech and narrative comprehension—that are present in *Raiders* but not in *Clips* (see Section Experimental Paradigms for more details). Contributions to these networks come largely from the *Auditory Association Cortex*, *Early Auditory Cortex*, *Temporo-Parieto-Occipital Junction*, *Posterior Cingulate Cortex*, *Parietal Cortex* and *Lateral Temporal Cortex* (see Supplementary Table 1).

#### Retinotopy study

**Fig. 6:**
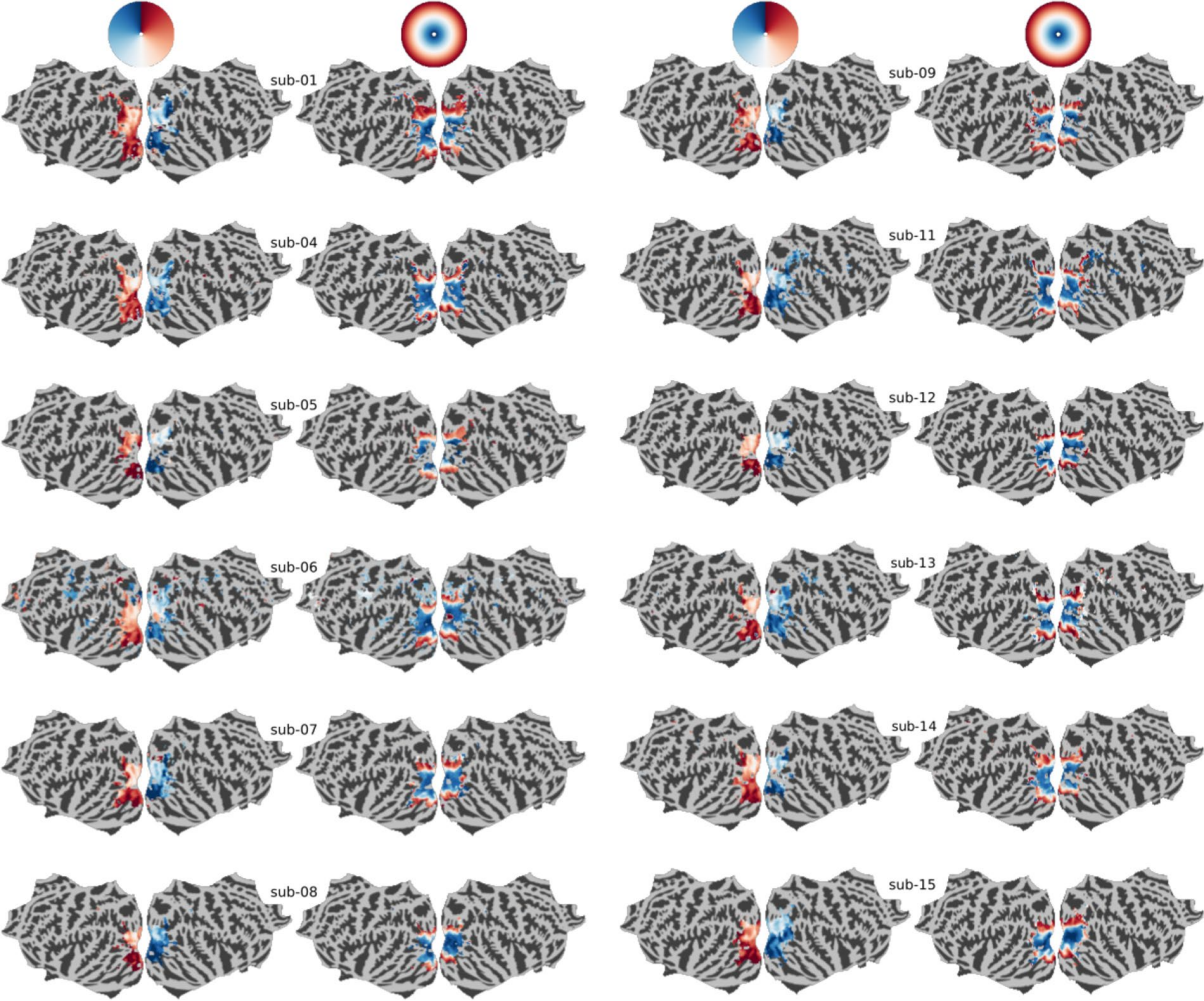
Individual, flat and binary maps of retinotopy in the visual field. (top) The visual field is encoded through polar coordinates: polar angle(left) and eccentricity (right). These polar coordinates are mapped on a flattened representation of the cortical surfaces extracted from the twelve IBC subjects: sub-01, sub-04, sub-05, sub-06, sub-07 and sub-08 on the left side; sub-09, sub-11, sub-12, sub-13, sub-14 and sub-15 on the other right side. One shall note the striking similarity of these maps across individuals. Individual binary maps for fixed effects are displayed for every participant, using an FDR-corrected threshold *q* = 0.05.

Figure 6 shows the retinotopic organization of the visual field in the human brain elicited by the *Retinotopy* tasks (see Sections Materials, Experimental Procedure, *Retinotopy* tasks for further details about the implementation of these classic retinotopy paradigms). The topographic projection to the V1-4 brain areas of the top-down and left-right reversed representation in the retina of the visual stimuli is mapped for every participant. As in Sereno *et al*.^32^, projections from the *Wedge* task *(left column)* represent the map of polar angle, whereas those from the *Ring* task *(right column)* represent the map of eccentricity. Overall, one can clearly notice a consistent spatial encoding of the visual field through these polar coordinates across all individuals.

In addition, we identified some areas of signal loss, for example in sub-05. After inspection, we concluded that this was probably due to signal correction in the most posterior part of the image during acquisition.

## Usage Notes

Our results show that functional-imaging data featuring the third release of the IBC dataset reflect response to behavior during performance of the corresponding tasks. We also show the feasibility of extracting the same type of data derivatives—i.e. contrast maps—from tasks pertaining to different types of experimental designs. Concretely, we demonstrate that cognitive networks of functional data collected from naturalistic paradigms can be extracted using FastSRM—an unsupervised data-driven method—without explicitly modelling features of the stimuli. Due to various shortcuts described in Richard&Thirion (2023)^34^, the fast and memory-efficient implementation of FastSRM becomes very useful under high-dimensional regimes, such as modelling naturalistic stimuli. In addition, we demonstrate that results obtained for every task–which are adapted from previous studies–are in agreement with the ones originally reported.

Data collection ended in October 2023 and final releases are planned for this year and next year. Tasks featuring these releases will comprise not only other sensory modalities in greater depth but also high-order cognitive modules that will complement those from past releases. For instance, we plan to attain a better coverage of the auditory system with the inclusion of tasks on tonotopy, auditory language comprehension and listening of naturalistic sounds. Other tasks on biological motion, motor inhibition, finger tapping, visual perception (e.g. color, scenes and faces), stimulus salience, working memory, emotional memory, spatial navigation, risk-based decision making, reward processing, language and arithmetic processing will also integrate these future releases.

Although the IBC dataset is dedicated foremost to task-fMRI data, future releases will be also dedicated to resting-state fMRI data as well as to other MRI modalities, concretely high-resolution T1- and T2-weighted, diffusion-weighted and myelin water fraction.

The official website of the IBC dataset (https://individual-brain-charting.github.io/docs) can be consulted anytime for a continuous update about its releases.

### Supplementary information

Supplementary Information

Supplementary material

Supplementary material

## Data Availability

Metadata concerning the stimuli presented during the BOLD fMRI runs are publicly available at https://github.com/individual-brain-charting/public_protocols. They include: *(1)* the behavioral protocols of the tasks; *(2)* the demo presentations of the tasks as video-demo presentations; *(3)* the instructions to the participants; and *(4)* the scripts to extract paradigm descriptors from log files for the GLM estimation. Regarding the behavioral protocols, *Clips* task consists in a reproduction of the protocol featuring the original study, only with minor adjustments and most of them concerned with experimental settings; *Retinotopy* and *Raiders* tasks were re-written from scratch in Python with no change of the design referring to the original paradigms. Regarding the demo presentations of the *Retinotopy* tasks, they can also be found on the YouTube channel of the IBC project: https://www.youtube.com/@individualbraincharting6314. The scripts used for data analysis are publicly available under the Simplified BSD license: https://github.com/individual-brain-charting/public_analysis_code. The API code of the IBC data-fetcher from EBRAINS is available on a public GitHub repository at https://github.com/individual-brain-charting/api. This public repository includes also information about installation and a minimal example usage.
