# Supplementary material for: Individual Brain Charting dataset extension, third release for movie watching and retinotopy data
Source: Sci Data. 2024 Jun 5;11:590. doi: 10.1038/s41597-024-03390-1 (PMC11153490; doi:10.1038/s41597-024-03390-1)
Supplement: Supplementary file 2 — Supplementary material [file 41597_2024_3390_MOESM2_ESM.pdf]

Description of the Supplementary Material

Pinho, A. L. et al.

May 13, 2024

1 Behavioral Scores of Training Sessions for Clips and Retinotopy tasks

File: **supplementary_material.pdf**

This material is mentioned in the Section "Data Acquisition: Behavioral Data" of the article.

Scores were obtained as percentages of response accuracy that measured how close participant's sight was from the center of the screen during the display of naturalistic scenes for the given trial. Each trial last for approximately one minute.

Higher scores mean that the participant consistently kept their eyes during this period fixed at a point closer to the center of the screen.

These scores are illustrative of the outcome obtained during the training sessions. We note that we opted to not always conduct this training because of the fatigue induced in the participants. We made sure however that every participant would be exposed at least once to the training.
