# Supplementary material for: Individual Brain Charting dataset extension, third release for movie watching and retinotopy data
Source: Sci Data. 2024 Jun 5;11:590. doi: 10.1038/s41597-024-03390-1 (PMC11153490; doi:10.1038/s41597-024-03390-1)
Supplement: Supplementary file 3 — Supplementary material [file 41597_2024_3390_MOESM3_ESM.pdf]

Session 1

Subjects/Scores	Trial1	Trial2	Trial3	Trial4
**sub-01**	-	-	-	-
**sub-02**	22	90	82	82
**sub-04**	16.83	92.67	91.33	98.08
**sub-05**	-	-	-	-
**sub-06**	40.5	88.67	69	75.33
**sub-07**	-	-	-	-
**sub-08**	8.08	35.33	97.92	85.67
**sub-09**	44.83	54.58	49.92	57.5
**sub-11**	59.5	32.33	56.83	90.58
**sub-12**	-	-	-	-
**sub-13**	20.58	41.68	67.58	97.75
**sub-14**	-	-	-	-
**sub-15**	39	82.58	68.5	46.17

Session 2

Subjects/Scores	Trial1	Trial2	Trial3	Trial4
**sub-01**	57.67	66.25	45.58	30.92
**sub-02**	66.92	99.5	99.3	98.5
**sub-04**	54.33	95	89	98.5
**sub-05**	20.42	85.33	35.92	35.75
**sub-06**	60.08	91.17	90.57	98.33
**sub-07**	-	-	-	-
**sub-08**	31.83	6.75	15	5.67
**sub-09**	3.83	6.42	1.25	55.5
**sub-11**	49.17	96.33	88.17	89.67
**sub-12**	-	-	-	-
**sub-13**	39.42	1.58	18.42	11.67
**sub-14**	38.75	19.33	94.67	47.25
**sub-15**	70.17	31.25	61.92	28.75

Session 3

Subjects/Scores	Trial1	Trial2	Trial3	Trial4
**sub-01**	-	-	-	-
**sub-02**	80	98.58	64	94.5
**sub-04**	65.17	69.17	12.75	7.58
**sub-05**	5.67	50.75	45.65	41.33
**sub-06**	-	-	-	-
**sub-07**	6.58	59.68	45	55.33
**sub-08**	-	-	-	-
**sub-09**	52	29.17	38.08	47.67
**sub-11**	48.08	72.48	37.5	25.58
**sub-12**	34.08	97.58	100	99.33
**sub-13**	-	-	-	-
**sub-14**	39.92	93.75	80.42	65.83
**sub-15**	66	54.58	87.75	90.52

Session 4

Subjects/Scores	Trial1	Trial2	Trial3	Trial4
sub-01	-	-	-	-
sub-02	70.75	96.17	86.92	69.83
sub-04				
sub-05	5.42	3.67	5.42	0.75
sub-06	89.25	96.17	92.92	97.58
sub-07	10.25	90.75	95	89.58
sub-08	50.5	85	82.42	99.17
sub-09	63.92	63.92	60.25	86.08
sub-11	47.75	70.33	70.25	82.33
sub-12	62.42	99.42	91.75	92
sub-13	-	-	-	-
sub-14	-	-	-	-
sub-15	39.75	33.58	41.67	2.75
